# Validation of the Taiwan Chinese version of the EORTC QLQ-CR29 to assess quality of life in colorectal cancer patients

**DOI:** 10.1186/s12885-018-4312-y

**Published:** 2018-04-02

**Authors:** Ming-Hung Shen, Ling-Ping Chen, Thien-Fiew Ho, Ying-Yih Shih, Ching-Shui Huang, Wei-Chu Chie, Chi-Cheng Huang

**Affiliations:** 1 Division of Colorectal Surgery, Department of Surgery, Cathay General Hospital, No.280, Jen-I Rd. Sec.4, Daan Dist., Taipei City, 106 Taiwan; 2 Division of General Surgery, Department of Surgery, Fu-Jen Catholic University Hospital, No.69; Gui-Zi Rd., Taishan Dist., New Taipei City, 243 Taiwan; 3 Division of Hematology and Oncology, Department of Medicine, Cathay General Hospital, No.280, Jen-I Rd. Sec.4, Daan Dist., Taipei City, 106 Taiwan; 4 Department of Surgery, Cathay General Hospital Sijhih, No.2, Lane 59, Jian-Cheng Rd., Sijhih Dist., New Taipei City, 221 Taiwan; 5 Department of Medicine, Cathay General Hospital Sijhih, No.2, Lane 59, Jian-Cheng Rd., Sijhih Dist., New Taipei City, 221 Taiwan; 6 Division of General Surgery, Department of Surgery, Cathay General Hospital, No.280, Jen-I Rd. Sec.4, Daan Dist., Taipei City, 106 Taiwan; 7 School of Medicine, College of Medicine, Taipei Medical University, No.250, Wu-Xing St., Xinyi Dist., Taipei City, 110 Taiwan; 8 Institute of Epidemiology and Preventive Medicine, College of Public Health, National Taiwan University, No.17, Xu-Zhou Rd., Zhongzheng Dist., Taipei City, 100 Taiwan; 9 School of Medicine, College of Medicine, Fu-Jen Catholic University, No.510, Zhong-Zheng Rd., Xinzhuang Dist., New Taipei City, 242 Taiwan

**Keywords:** Colorectal cancer, Quality of life, EORTC QLQ-C30 and QLQ-CR29, Validation study, Taiwan Chinese

## Abstract

**Background:**

The increasing incidence of colorectal cancer in Taiwan has generated a need for a disease-specific quality-of-life measuring instrument. We aimed to validate the Taiwan Chinese version of the European Organisation for Research and Treatment of Cancer (EORTC) QLQ-C30 and QLQ-CR29.

**Methods:**

A total of 108 patients were interviewed. Convergent and discriminant validity, Cronbach’s alpha coefficient, test-retest reliability, and known-groups comparisons were used to examine the reliability and validity.

**Results:**

We found good internal consistency reliability for multi-item scales of the QLQ-C30 and QLQ-CR29, except for the cognitive function and pain scale of the QLQ-C30. Patients in the active treatment group reported compromised functional scale scores (global health status/quality of life, QLQ-C30) and worse symptoms (blood and mucus in stool, QLQ-CR29) than those in the follow-up group. Similar results were found in comparisons based on Eastern Cooperative Oncology Group (ECOG) Performance Status and Bristol Stool Scale: higher physical function/sexual interest, less fatigue/urine frequency symptoms for patients with the lowest ECOG Performance Status (Grade 0), and borderline worse stool frequency scores from Types 5 and 6 patients on the Bristol Stool Scale.

**Conclusion:**

The study validated the Taiwan Chinese version of the EORTC QLQ-C30 and QLQ-CR29. The clinical applicability warrants further studies with greater number of participants.

## Background

The concepts of quality of life and patient-centered outcomes have become popular in medical communities; however, the wide application of quality-of-life investigations remains an obstacle for most clinicians due to the limited validation studies performed to date and the lack of disease-specific measuring instruments. Health-related quality of life has become an indispensable component of outcomes research, particularly for cancer therapy. Measurement instruments, particularly self-administrated questionnaires, enable a quantitative approach to the multi-dimensional perception of quality of life, and such surveys may provide important outcome variables in addition to conventional clinical results such as morbidity and disease-free survival [1].

Colorectal cancer is the leading cause of human malignancies in Taiwan according to the Bureau of Health Promotion, and ranks the third among all cancer deaths [2]. The burden of colorectal cancer is rapidly increasing due to the high incidence and consequences of cancer therapy. Patients who survived colorectal cancer therapies may continue to suffer from physical or psychological problems [3]. For example, chemotherapy may hamper quality of life considerably, and colon/rectum resection may result in long-term prolonged diarrhea or fecal incontinence. Therefore, development and validation of a measuring instrument is an urgent requirement for medical professionals and cancer patients.

The European Organisation for Research and Treatment of Cancer (EORTC) QLQ-CR29 [4, 5] is a colorectal cancer-specific module supplementary to the core quality-of-life questionnaire QLQ-C30 [6]. However, the validity and reliability of the Taiwan Chinese version have never been conducted, and only the early results had been reported in Mainland China using the Simplified Chinese version, which is distinct from the Traditional Chinese version used in Taiwan [7]. The present study aimed to assess the reliability and validity of the EORTC QLQ-C30 and QLQ-CR29 for patients with colon and rectal cancer in Taiwan.

## Methods

### Translation of the Taiwan Chinese version of the EORTC QLQ-CR29

Traditional Chinese (Mandarin) language used in Taiwan is linguistically different from the Simplified Chinese used in Mainland China. The translation and pilot study were conducted during the years 2007 and 2008, shortly after the introduction of the updated English version of colorectal cancer-specific module QLQ-CR29. Fifty-seven Taiwanese patients were enrolled as part of the multi-national validation study [5]. The Taiwan Chinese EORTC QLQ-CR29 was developed using a standard procedure of translation and back-translation [8], after which the questionnaire was reviewed and approved by the EORTC Quality of Life Group.

### Study population

Patient recruitment began on November 1, 2015 and ended on March 31, 2016 at Cathay General Hospital. Patients over 18 years of age with pathology-proved colon or rectum cancer were invited during the enrollment period. Patients’ status was categorized into the active treatment or follow-up group. Pre-operative patients or patients under chemotherapy constituted the active treatment group, and these patients were interviewed before surgery or after the first day of chemotherapy. Follow-up patients were those who had completed surgery, chemotherapy, or any adjuvant therapy for at least six months, and their interviews were conducted during returning visits at outpatient clinics. Exclusion criteria included disagreement to participate, concurrent secondary malignancy, concurrent engagement in another quality-of-life study, and declaration of critical illness. Study purpose and privacy protection policy were effectively explained with written consent obtained from all participants.

### Measuring instruments

The EORTC QLQ-C30 core questionnaire is a quality-of-life measuring instrument for cancer patients, and the Taiwan Chinese (Traditional Chinese) version has been validated and descripted previously [9, 10]. The clinical applicability for breast cancer, lung cancer, head and neck cancer, gastric cancer, and esophageal cancer has been demonstrated [9–13]. The QLQ-C30 consists of a global health status/quality of life, five multi-item functional scales and several multi-item symptomatic scales or single items. With linear transformation, seven- and four-level Likert scales (seven for the global health status/quality of life scale and four for the others) were converted to a 0 to 100 score with 100 representing the best global health, functional status, or worst symptom depending on the measuring characteristic of each multi-item scale or single item [14].

The EORTC QLQ-CR29 is a 29-item colon and rectum cancer site-specific supplemental module that aims to enhance the sensitivity and specificity for colorectal cancer quality of life measures [4, 5]. The original English version comprises 4 multi-item scales (body image, urinary frequency, blood and mucus in stool, and stool frequency) and 17 functional/symptomatic single-items (anxiety, weight, sexual interest, urinary incontinence, dysuria, abdominal pain, buttock pain, bloating, dry mouth, hair loss, taste, flatulence, fecal incontinence, sore skin, embarrassment, stoma care problem, impotence or dyspareunia), with higher scores indicating better functional or worse symptomatic status. Of these 21 scales or items, only body image, anxiety, weight, and sexual interest are functional domain scales/items, and all the remaining are symptomatic. One item (Q18) of the QLQ-CR29 is an indicator of colostomy/ileostomy construction, and different contents are designed for patients with/without a stoma in stool frequency, flatulence, fecal incontinence, sore skin, and embarrassment. Separate items are arranged for patients with a stoma (Q19-Q25) and those without it (Q19-Q24). The stoma care problem is only eligible to patients with a colostomy/ileostomy (Q25). Moreover, sexual interest, impotence, and dyspareunia items are only applicable to the corresponding gender (Q26-Q27 for male and Q28-Q29 for female). Permission to use the QLQ-C30 and QLQ-CR29 was obtained in advance from the EORTC Quality of Life Department.

### Additional measures

Additional measures were rated by two investigators (MHS and CCH, both of who are qualified colorectal surgeons) to assess patients’ performance status and colonic transit time in the week prior to administering the questionnaires. Eastern Cooperative Oncology Group (ECOG) Performance Status, evaluates a patient’s level of functioning, and is widely used in cancer research, with Grade 0 representing fully active and Grade 5 representing dead status [15]. Bristol Stool Scale is adopted from Lewis et al. [16], which categorizes the form of stool representing colonic transit time. In brief, Type 1 and 2 indicate stool constipation, while Type 5-7 indicate diarrhea.

### Reliability and validity

Internal consistency reliability was evaluated for multi-item scales, and a referable reliability was indicated by Cronbach’s alpha coefficient greater than 0.70 [17]. For multi-item scales, both convergent and discriminant validity were evaluated by item-scale correlations. Convergent validity was indicated by item and item-own scale correlation greater than 0.40, and item and item-own scale correlation greater than item-other scale correlations demonstrated discriminant validity [18]. A subset of follow-up patients was re-assessed within 7-14 days for the test-retest reliability (reproducibility) by evaluating the correlation coefficients between repeated measures during December 2017.

Known-groups comparisons, which compared patients of different treatment conditions, ECOG Performance Status, and Bristol Stool Scale, were conducted for the purpose of evaluating clinical validity. We postulated that patients under active treatment may suffer from disease burden or treatment adverse effects, and higher symptomatic and lower functional scores were discernable. Patients with higher degree of diarrhea according to the Bristol Stool Scale may have worse diarrhea-related symptoms, and patients with better ECOG Performance Status may report higher functional and lower symptomatic scores. Additional comparisons regarding the presence of a stoma, the type of adjuvant therapy, and different surgical procedures were evaluated as well.

### Statistical analysis

Wilcoxon rank sum test was used for comparing group means since most quality-of-life scores were skewed and not normally distributed. All tests were two-sided, and a *P*-value less than 0.05 was considered as statistically significant. Sample size was calculated by G*Power3 [19] and was estimated as follows: assuming the standard deviation was 20, in order to detect a difference of 10 to 15 scores between two groups, the number needed in each group was 51 and 23, respectively, under the two-sided Z test with 80% power and α level of 0.05. Consequently a total of 50 patients in each group were a prerequisite for the validation purpose. The presuming quality-of-life score difference as well as standard deviation were estimated from our previous validation study for the QLQ-BR23, QLQ-STO22, and the suggestion of Osoba et al. [9, 10, 20].

## Results

### Demographic features

During the enrollment period, 108 colorectal cancer patients (53 from the active treatment and 55 from the follow-up group) were successfully interviewed. There were 63 males and 45 females, with the mean age being 63.7 years (range: 22.2~ 89.1, SD: 13.2). Among them, 20 (18.5%) patients were presented with an obstructive lesion during initial diagnosis. The response rate was 88% for the active treatment and 87% for the follow-up group (refusers: 7 for active treatment and 8 for the follow-up group), with no significant difference (Fisher’s exact test, *P* = 0.21). Of the 53 patients in active treatment, 20 were planned for surgery and 33 for chemotherapy. Descriptive statistics are listed in Table 1. There was no difference in demographic and clinical features except more female patients in the follow-up group, and more stage IV and chemotherapy patients in the active treatment group (*P* < 0.05). There was no difference in terms of the ECOG Performance Status and the Bristol Stool Scale between these two groups. The distributions of the EORTC QLQ-C30 and QLQ-CR29 scale scores are detailed in Table 2.

**Table 1:** Demographic and clinical features of study population

Group	Active treatment	Follow-up^a^	P-value^*^
Case number	53	55	
Male:female	37:16	26:29	***P*** **= 0.02**
Age^d^	61.5(12.5)	65.8(13.8)	*P* = 0.10
Marriage			
Married:single	37:16	42:12	*P* = 0.40
Education			
Illiterate	1	3	*P* = 0.27
Elementary/junior high school	13	21	
High school	20	17	
College/University/Graduate school	19	13	
Occupation			
Full/part time	26	18	*P* = 0.16
Retired	27	36	
ECOG status score			
0	39	41	*P* = 0.20
1	11	5	
2	2	7	
3	1	1	
Bristol Stool Scale			*P* = 0.22
1	1	0	
2	2	1	
3	3	5	
4	25	17	
5	16	16	
6	6	15	
Tumor site			
A−/T-colon	15	11	*P* = 0.69
D-colon	6	5	
Sigmoid colon	14	18	
Rectum/anus	18	20	
Stage			
0 (in situ)	2	4	***P < 0.01***
I	4	18	
II	11	13	
III	21	17	
IV	15	2	
Surgery			
None	4^b^	0	***P = 0.05***
Minimally invasive	25	36	
Laparotomy	24	18	
Stoma			
None	46	51	*P* = 0.31
Colonostomy/ileostomy	7	4	
Chemotherapy			
With:without	42:11^c^	27:27	***P < 0.01***
Radiotherapy			
With:without	9:44	5:49^a^	*P* = 0.47
Obstructive lesion	13	7	*P* = 0.11

^*^χ^2^-test for categorical variables and Kruskal-Wallis test for continuous variables. *P*-values in boldface indicate significant between-group difference

^a^One missing value in the follow-up group

^b^One patient did not undergo planned surgery

^c^Pre-operative(neo-adjuvant) chemotherapy

^d^Age presented as mean (standard deviation)

**Table 2:** Distributions of the EORTC QLQ-C30 and QLQ-CR29 scores for Taiwanese colorectal cancer patients

Scale	*N*	Mean	Standard deviation	Minimum	Maximum	Reproducibility
QLQ-C30						
QL2(Q29-Q30)	102	63.9	21.3	0.0	100.0	0.82
PF(Q1-Q5)	108	81.9	19.0	20.0	100.0	0.81
RF(Q6-Q7)	107	79.6	25.6	0.0	100.0	0.84
EF(Q21-Q24)	106	80.9	17.7	25.0	100.0	0.64
CF(Q20,Q25)	106	79.7	18.0	33.3	100.0	0.48
SF(Q26,Q27)	106	78.1	22.5	0.0	100.0	0.58
FA(Q10,Q12,Q18)	107	30.4	20.2	0.0	88.9	0.72
NV(Q14,Q15)	108	8.2	13.8	0.0	66.7	0.52
PA(Q9,Q19)	108	17.1	17.6	0.0	83.3	0.11
DY(Q8)	107	13.1	19.3	0.0	100.0	0.29
SL(Q11)	106	24.5	26.5	0.0	100.0	0.74
AP(Q13)	108	16.4	23.0	0.0	100.0	0.59
CO(Q16)	107	24.3	26.1	0.0	100.0	0.56
DI(Q17)	106	23.3	29.2	0.0	100.0	0.63
FI(Q28)	106	15.1	23.5	0.0	100.0	0.47
QLQ-CR29						
BI(Q45-Q47)	105	86.7	19.2	0.0	100.0	0.89
ANX(Q43)	105	62.9	24.2	0.0	100.0	0.47
WEI(Q44)	105	74.6	24.3	0.0	100.0	0.48
SEXF(Q56,Q58)	99	17.5	19.8	0.0	100.0	0.47
UF(Q31,Q32)	105	21.6	21.4	0.0	100.0	0.51
BMS(Q38,Q39)	104	11.7	19.1	0.0	100.0	0.34
SF2(Q52,Q53)	99	20.7	20.8	0.0	83.3	0.78
UI(Q33)	105	6.3	17.4	0.0	100.0	0.11
DY2(Q34)	105	2.5	8.9	0.0	33.3	0.69
AP2(Q35)	105	14.0	21.6	0.0	100.0	0.68
BP(Q36)	103	10.0	17.4	0.0	66.7	0.71
BF(Q37)	104	20.5	22.9	0.0	100.0	0.40
DM(Q40)	105	28.9	20.2	0.0	100.0	0.09
HL(Q41)	103	17.5	28.7	0.0	100.0	0.85
TA(Q42)	104	11.2	21.6	0.0	100.0	0.67
FL(Q49)	101	21.8	26.0	0.0	100.0	0.81
FI2(Q50)	101	10.6	20.0	0.0	100.0	0.47
SS(Q51)	98	8.2	16.6	0.0	66.7	0.63
EMB(Q54)	100	13.7	24.2	0.0	100.0	0.50
STO(Q55)	13	5.1	12.5	0.0	33.3	0.89
IMP(Q57)	59	20.3	21.5	0.0	100.0	0.97
DYS(Q59)	39	9.4	20.2	0.0	66.7	0.65

*QL2* global health/quality of life, *PF* physical function, *RF* role function, *EF* emotion function, CF cognitive function, *SF* social function, *FA* fatigue, *NV* nausea/vomiting, *PA* pain, *DY* dyspnea, *SL* insomnia, *AP* appetite loss, *CO* constipation, *DI* diarrhea, *FI* financial difficulty, *BI* body image, *ANX* anxiety, *WEI* weight, *SEXF* sexual interest, *UF* urinary frequency, *BMS* blood and mucus in stool, *SF2* stool frequency, *UI* urinary incontinence, *DY2* dysuria, *AP2* abdominal pain, *BP* buttock pain, *BF* bloating, *DM* dry mouth, *HL* hair loss, *TA* taste, *FL* flatulence, *FI2* faecal incontinence, *SS* sore skin, *EMB* embarrassment, *STO* stoma care problem, *IMP* importance, *DYS* dyspareunia

### Reproducibility

Table 2 also displays reproducibility (test-retest reliability) for multi−/single- item scales of the EORTC QLQ-C30 and QLQ-CR29. A subset of 30 follow-up patients were approached, and 20 completed repeated measures between the first and second assessments within 7-14 days. Most scales indicated moderate to high correlation coefficients (0.51-1), augmenting the reproducibility of the measuring instruments. Exceptions were cognitive function (*r* = 0.48), pain (*r* = 0.11), dyspnea (*r* = 0.29), and financial difficulty (*r* = 0.47) from the QLQ-C30, as well as anxiety (*r* = 0.47), weight (*r* = 0.48), sexual interest (*r* = 0.47), blood and mucus in stool (*r* = 0.34), urine incontinence (r = 0.11), bloating (*r* = 0.40), dry mouth (*r* = 0.09), fecal incontinence (*r* = 0.47), and embarrassment (*r* = 0.50) scales from the QLQ-CR29. It is noteworthy that test-retest reliability was performed for the same follow-up group separately during December 2017.

### Reliability

Table 3 exhibited the reliability of the Taiwan Chinese version of the EORTC QLQ-C30 and QLQ-CR29. Convergent validity was indicated by item-own scale correlation (corrected for overlap) above 0.40 for all multi-item scales, and discriminant validity was convinced as the item own scale correlation was higher than item-other scale correlations for all multi-item scales. Cronbach’s alpha coefficient indicated good internal consistency reliability (> 0.70) for the QLQ-C30 and the QLQ-CR29 except cognitive function (0.45) and pain (0.61), both of which were from the QLQ-C30.

**Table 3:** Reliability, convergent and discriminative validity of the Taiwan Chinese EORTC QLQ-C30 and QLQ-CR29

Scale	Convergent validity	Discriminative validity	Cronbach’s α
QLQ-C30 function			
QL2	0.95-0.96	0.27-0.54	0.88
PF	0.57-0.87	0.06-0.49	0.82
RF	0.96-0.97	0.12-0.54	0.93
EF	0.70-0.86	0.10-0.52	0.8
CF	0.78-0.82	0.07-0.45	0.45
SF	0.85-0.91	0.09-0.52	0.73
QLQ-C30 Symptom			
FA	0.86-0.87	0.12-0.41	0.83
NV	0.86-0.93	0.13-0.32	0.75
PA	0.84	0.01-0.42	0.61
QLQ-CR29 function			
BI	0.87-0.90		0.9
QLQ-CR29 symptom			
UF	0.91	0.04-0.32	0.82
BMS	0.88	0.01-0.37	0.78
SF2	0.84-0.91	0.27-0.35	0.7

*QL2* global health/quality of life, *PF* physical function, *RF* role function, *EF* emotion function, CF cognitive function, *SF* social function, *FA* fatigue, *NV* nausea/vomiting, *PA* pain, *BI* body image, *UF* urinary frequency, *BMS* blood and mucus in stool, *SF2* stool frequency

### Clinical validity

Table 4 presented the results of clinical validity. Follow-up patients reported a higher functional score in global health status/quality of life than those undergoing active treatment (*P* = 0.005). On the other hand, worse blood and mucus in stool was reported by patients in the active treatment group (19 versus 4, *P* < 0.001). The EORTC QLQ-CR29 recognized this as a colorectal cancer-specific symptom.

**Table 4:** Clinical validity: known-group comparisons of the Taiwan Chinese EORTC QLQ-C30 and QLQ-CR29

Scale	Treatment			ECOG Status Score			Bristol Stool Scale		
	Active treatment	Follow-up	*P*-value^&^	ECOG = 0	ECOG = 1,2,3	*P*-value^&^	BSS = 0,1,2,3,4	BSS = 5,6	*P*-value^&^
	*n* = 53	*n* = 55		*n* = 81	*n* = 27		*n* = 55	*n* = 53	
QLQ-C30 function									
QL2	57.5	70.3	**0.005**	66.6	54.7	**0.018**	61.4	66.5	0.279
PF	83.5	80.3	0.233	87.3	67.3	**0.000**	81.9	81.8	0.774
RF	75.5	83.6	0.112	84.0	68.5	**0.015**	77.5	81.8	0.544
EF	81.0	80.8	0.783	81.5	78.7	0.384	79.9	81.9	0.821
CF	81.1	78.3	0.529	81.3	74.7	0.113	77.8	81.7	0.264
SF	74.5	81.8	0.081	80.4	71.3	0.062	74.1	82.4	**0.049**
QLQ-C30symptom									
FA	31.7	29.1	0.717	26.4	42.5	**0.001**	31.1	29.6	0.698
NV	9.4	7.0	0.570	8.1	8.6	0.958	10.3	6.0	0.064
PA	18.2	16.1	0.734	15.0	24.1	**0.020**	18.5	15.7	0.400
DY	10.9	15.2	0.306	12.5	15.4	0.278	11.1	15.1	0.359
SL	21.4	27.7	0.321	23.6	26.9	0.836	23.0	26.1	0.512
AP	21.4	11.5	0.063	13.8	24.7	**0.041**	21.2	11.3	**0.034**
CO	23.3	25.3	0.601	21.1	33.3	**0.032**	22.2	26.4	0.310
DI	25.2	21.4	0.881	24.6	20.0	0.637	24.7	21.8	0.476
FI	15.1	15.1	0.910	15.4	13.3	0.996	17.3	12.8	0.334
QLQ-CR29 function									
BI	86.5	86.8	0.819	86.2	88.9	0.693	87.7	85.6	0.359
ANX	58.3	67.3	0.061	63.7	60.0	0.490	62.3	63.4	0.948
WEI	75.6	73.6	0.691	73.4	78.7	0.427	76.5	72.5	0.354
SEXF	15.6	19.3	0.444	21.5	5.3	**0.000**	15.3	19.7	0.446
QLQ-CR29 symptom									
UF	22.8	20.4	0.781	17.3	34.0	**0.001**	22.8	20.3	0.644
BMS	19.3	4.4	**0.000**	11.1	14.0	0.661	13.5	9.8	0.744
SF2	22.2	19.3	0.852	19.9	23.3	0.796	21.7	19.7	0.850
UI	5.1	7.5	0.971	6.3	5.3	0.842	5.6	7.2	0.750
DY2	3.2	1.9	0.451	3.0	1.3	0.434	3.7	1.3	0.169
AP2	13.5	14.5	0.426	14.3	13.3	0.479	11.7	16.3	0.110
BP	12.0	8.2	0.277	9.3	13.0	0.865	12.2	7.8	0.203
BF	21.2	19.9	0.980	20.5	20.0	0.612	18.5	22.7	0.213
DM	31.4	26.4	0.205	28.3	30.7	0.529	27.2	30.7	0.267
HL	19.2	15.7	0.469	18.6	14.7	0.450	19.5	15.3	0.950
TA	9.6	12.8	0.596	13.2	5.3	0.133	11.3	11.1	0.916
FL	21.3	22.2	0.866	20.4	26.7	0.346	17.3	26.5	0.059
FI2	8.7	12.4	0.390	12.0	6.7	0.204	10.3	10.9	0.901
SS	9.7	6.7	0.389	6.5	13.3	0.123	8.8	7.5	0.514
EMB	14.3	13.1	0.616	13.1	16.0	0.516	11.8	15.6	0.514
STO	11.1	0.0	0.138	3.3	11.1	0.418	0.0	6.7	0.500
IMP	17.1	25.0	0.100	18.9	23.8	0.894	21.5	19.0	1.000
DYS	11.9	8.0	0.417	12.6	0.0	0.071	9.3	9.5	0.889

*QL2* global health/quality of life, *PF* physical function, *RF* role function, *EF* emotion function, CF cognitive function, *SF* social function, *FA* fatigue, *NV* nausea/vomiting, *PA* pain, *DY* dyspnea, *SL* insomnia, *AP* appetite loss, *CO* constipation, *DI* diarrhea, *FI* financial difficulty, *BI* body image, *ANX* anxiety, *WEI* weight, *SEXF* sexual interest, *UF* urinary frequency, *BMS* blood and mucus in stool, *SF2* stool frequency, *UI* urinary incontinence, *DY2* dysuria, *AP2* abdominal pain, *BP* buttock pain, *BF* bloating, *DM* dry mouth, *HL* hair loss, *TA* taste, *FL* flatulence, *FI2* faecal incontinence, *SS* sore skin, *EMB* embarrassment, *STO* stoma care problem, *IMP* importance, *DYS* dyspareunia

^&^Wilcoxon rank sum test. P-values in boldface indicate significant between-group difference in quality of life scores

Quality of life scores presented as medium (interquartile range)

When ECOG Performance Status was used for partitioning, better global health status/quality of life (*P* < 0.05, QLQ-C30); better role function (*P* < 0.05, QLQ-C30); better physical function (*P* < 0.01, QLQ-C30); improved sexual interest (*P* < 0.01, QLQ-CR29); fewer complaints of fatigue (*P* < 0.01, QLQ-C30); lesser constipation, pain, and appetite loss (*P* < 0.05, QLQ-C30); and lesser problems with urinary frequency (*P* < 0.01, QLQ-CR29) were observed for ECOG Performance Status Grade 0 patients compared with patients having Grades 1-3. Types 5 and 6 patients on the Bristol Stool Scale experienced less appetite loss, and had better social function compared to Types 1 to 4 patients on the Bristol Stool Scale (*P* < 0.05, QLQ-C30).

Further comparisons evaluating the impacts of colostomy/ileostomy construction, adjuvant therapy, and surgical methods upon quality of life are detailed in Table 5. Stoma construction inevitably hampered quality of life in sore skin and fecal incontinence (*P* < 0.05, QLQ-CR29), while less insomnia (*P* < 0.05, QLQ-C30) was also revealed for the stoma group. Minimally invasive surgery benefited colorectal cancer patients with better social function, and fewer buttock pain and nausea/vomiting symptoms (*P* < 0.05). Adjuvant therapy deteriorated quality of life with worse hair loss and compromised social function (*P* < 0.01).

**Table 5:** Additional known-group comparisons of the Taiwan Chinese EORTC QLQ-C30 and QLQ-CR29

Scale	Stoma			Surgery			Adjuvant therapy		
	Without stomy	With stomy	*P*-value^&^	Minimally invasive	Laparotomy	*P*-value^&^	Without adjuvant therapy	With adjuvant therapy	*P*-value^&^
	*n* = 97	*n* = 11		*n* = 61	*n* = 42		*n* = 38	*n* = 65	
QLQ-C30 function									
QL2	64.1	62.1	0.706	64.3	66.0	0.793	65.7	62.9	0.610
PF	81.9	81.8	0.516	83.0	82.2	0.447	84.3	80.5	0.143
RF	80.7	69.7	0.095	82.8	78.9	0.315	84.2	77.0	0.137
EF	79.9	89.4	0.090	80.6	83.7	0.482	84.5	79.0	0.136
CF	78.6	89.4	0.059	78.9	82.5	0.473	81.5	78.7	0.514
SF	79.1	69.7	0.182	83.6	72.8	**0.012**	86.0	73.9	**0.009**
QLQ-C30 symptom									
FA	30.8	26.3	0.591	31.1	27.6	0.805	27.3	32.0	0.353
NV	7.4	15.2	0.124	5.2	11.5	**0.028**	4.7	10.1	0.064
PA	16.7	21.2	0.521	15.8	17.9	0.633	17.5	16.9	0.871
DY	13.2	12.1	0.995	13.1	11.4	0.977	12.3	13.5	0.966
SL	26.3	9.1	**0.034**	28.2	19.8	0.161	27.9	22.7	0.400
AP	16.2	18.2	0.963	14.2	16.7	0.897	12.0	18.8	0.114
CO	25.0	18.2	0.569	26.2	22.8	0.625	25.6	23.5	0.811
DI	22.8	27.3	0.380	25.0	17.1	0.380	23.4	23.2	0.830
FI	15.1	15.2	0.662	12.2	16.3	0.272	10.8	17.4	0.124
QLQ-CR29 function									
BI	86.4	88.9	0.908	88.3	85.4	0.455	89.8	84.9	0.094
ANX	62.1	69.7	0.377	62.7	65.9	0.513	62.3	63.2	0.880
WEI	75.2	69.7	0.289	74.6	75.6	0.969	78.9	72.1	0.103
SEXF	17.8	15.2	0.542	16.7	18.4	0.380	20.6	15.9	0.311
QLQ-CR29 symptom									
UF	21.1	25.8	0.498	20.9	20.3	0.852	21.1	21.9	0.933
BMS	11.6	12.1	0.712	10.6	9.8	0.792	11.8	11.6	0.923
SF2	19.1	33.3	0.053	19.6	19.4	0.878	24.3	18.5	0.067
UI	6.4	6.1	0.808	5.1	6.5	0.133	3.5	8.0	0.297
DY2	2.8	0.0	0.322	1.7	3.3	0.375	0.9	3.5	0.151
AP2	13.8	15.2	0.866	13.0	13.0	0.668	14.9	13.4	0.990
BP	8.7	21.2	0.089	6.9	14.2	**0.040**	7.2	11.6	0.173
BF	20.1	24.2	0.447	19.5	18.7	0.606	25.4	17.7	0.111
DM	28.7	30.3	0.664	28.2	27.6	0.940	24.6	31.3	0.100
HL	17.8	15.2	0.690	14.4	23.3	0.108	4.5	24.7	**0.000**
TA	10.8	15.2	0.891	9.0	15.8	0.100	5.4	14.4	0.056
FL	21.9	21.2	0.928	21.8	20.2	0.737	22.5	21.4	0.913
FI2	9.3	21.2	**0.025**	9.8	8.8	0.944	8.1	12.0	0.654
SS	6.5	21.2	**0.025**	7.0	8.1	0.777	5.6	9.7	0.181
EMB	12.7	21.2	0.089	14.4	9.9	0.685	9.9	15.9	0.275
STO		15.0		0.0	4.8	0.499	0.0	5.6	0.831
IMP	21.8	9.5	0.142	20.0	20.5	0.691	19.3	20.8	0.708
DYS	9.5	8.3	0.921	7.7	12.1	0.450	2.4	13.3	0.119

*QL2* global health/quality of life, *PF* physical function, *RF* role function, *EF* emotion function, CF cognitive function, *SF* social function, *FA* fatigue, *NV* nausea/vomiting, *PA* pain, *DY* dyspnea, *SL* insomnia, *AP* appetite loss, *CO* constipation, *DI* diarrhea, *FI* financial difficulty, *BI* body image, *ANX* anxiety, *WEI* weight, *SEXF* sexual interest, *UF* urinary frequency, *BMS* blood and mucus in stool, *SF2* stool frequency, *UI* urinary incontinence, *DY2* dysuria, *AP2* abdominal pain, *BP* buttock pain, *BF* bloating, *DM* dry mouth, *HL* hair loss, *TA* taste, *FL* flatulence, *FI2* faecal incontinence, *SS* sore skin, *EMB* embarrassment, *STO* stoma care problem, *IMP* importance, *DYS* dyspareunia

^&^Wilcoxon rank sum test. P-values in boldface indicated significant between-group difference in quality of life scores

Quality of life scores presented as medium (interquartile range)

## Discussion

During the past decade, the Taiwan Chinese version of the EORTC QLQ-C30 (3.0) and the breast (QLQ-BR23), head and neck (QLQ-HN35), stomach (STO22), lung (QLQ-LC13), and esophageal (QLQ-OES18) cancer-specific modules have shown good acceptability for Taiwanese cancer patients [9–13]. This is not the case of the EORTC colon and rectum-specific module. The QLQ-CR29 is the revised and shorter version of the QLQ-CR38 [21], with the Chinese version validated and reported in Hong Kong [22] and Mainland China [23]. The QLQ-CR38 questionnaire was limited in terms of missing data and lack of specificity, particularly with regard to emerging new technologies such as pre-operative chemo-radiotherapy, ultra-low anterior resection, and minimally invasive surgery [4]. The initial 6 scales and 11 items construct of the QLQ-CR29 was reformatted into the final structure of 4 scales and 17 items. Thaysen et al. have summarized that EORTC QLQ-CR29 contains 17 unchanged questionnaire items from the QLQ-CR38, 5 reworded items, and 7 new items [24].

The present study may be the first validation study of the Taiwan Chinese QLQ-CR29 questionnaire. Most multi-item scales exhibited adequate internal consistency reliability. The only two exceptions were cognitive function and pain scale of the QLQ-C30. Cronbach’s alpha of cognition function was much lower than 0.70, and compromised coefficients were also noted when Taiwanese breast, lung, gastric, and head and neck cancer patients were approached [9–12]. We have suggested that elimination of cognitive function may enhance the conceptual structure of the Taiwan Chinese version of the EORTC QLQ-C30 in the higher-order formative health-related quality of life model [25]. All item and item-own scale correlations (corrected for overlap) were greater than 0.40 and all item-own scale correlations were greater than item-other scale correlations, and satisfactory discriminant and convergent validities for both the QLQ-C30 and QLQ-CR29 were evidenced.

For clinical validity, we hypothesized that pre-operative patients were negatively affected by the colorectal lesion, and patients with chemotherapy experienced worse quality of life from treatment side effects or psychological distress. For example, worse blood and mucus in stool complaint in the active treatment group was compatible with concurrent disease burden. Follow-up patients reported a higher global heath/quality-of-life score, demonstrating good recovery after completion of cancer therapy. Better functions and fewer symptoms, including sexual interest and urine frequency of the QLQ-CR29, among patients with the lowest ECOG Performance Status (Grade 0) also suggested convincing clinical validity. It is noteworthy that Types 5 and 6 patients on the Bristol Stool Scale experienced more flatulence with a borderline significance (*P* = 0.059, Table 5). Additional comparisons identified worse hair loss and social function from adjuvant therapy as well as worse sore skin and fecal incontinence from colostomy/ileostomy. Interestingly, patients with a stoma reported a lower insomnia symptomatic score. Our study also revealed that minimally invasive surgery might benefit patients with better social function, and less buttock pain, and nausea/vomiting symptoms.

During the validation of the Dutch QLQ-CR29, Stiggelbout et al. suggested decreasing the number of single items, improving the scales, and increasing the reliability of the entire questionnaire [26]. Indeed, the number of scales/items displaying a significant difference between the active treatment and follow-up group had significantly reduced compared with that of the Taiwan Chinese QLQ-STO22 validation study [10]. The QLQ-STO22, which is seven items shorter than the QLQ-CR29, contains 5 multi-item scales and 3 single items while the QLQ-CR29 is composed of 4 multi-item scales and 17 single items. The significantly higher proportion of single items (59% versus 14% or 17/29 versus 3/22, compared to the QLQ-STO22) of the colorectal module may limit its ability to detect all minute differences under high dimensionality, raising concerns about sensitivity loss for single-item measures, and the problem of an excessive number of single items substantially compromising the measuring performance of the QLQ-CR29.

The current study has some limitations. First, our modest sample size may have resulted in compromised statistical power, considering that QLQ-CR29 is arranged with significantly greater number of single-item than multi-item scales, and inadequate sample size may result in fewer detected differences. For example, up to 16 distinguished multi−/single-item scales were observed in the original international validation study involving 351 participants with three rounds of known-group comparisons, but only one multi- and two single-item scales were discriminative when the Polish QLQ-CR29 was validated with an extremely compromised sample size of 20 [5, 27]. The yield of known-group analysis is largely influenced by the characteristics of the targeted population, stratification factor, as well as the number of colorectal cancer patients enrolled; a survey of 108 participants may just fulfil the purpose of a validation study, but are inadequate to detect all quality-of-life fluctuations across broad clinical scenarios. The clinical applicability of the QLQ-CR29 will be evaluated when more samples are enrolled in the future.

Second, reproducibility (test-retest reliability) was not conducted at the time when enrolled patients were initially contacted but was performed one year later. The non-concurrent, add-on design might hamper comparability and efficiency, and inevitably compromise reproducibility. It is noteworthy that the agreement in anxiety was not maintained when the Bahasa Malaysia version of the QLQ-CR29 was evaluated for test-retest correlations either [28].

## Conclusion

The validity and reliability of the Taiwan Chinese EORTC QLQ-C30 and QLQ-CR29 questionnaire were ascertained. Quality-of-life investigation is complimentary to traditional outcomes such as morbidity and mortality, while patients’ perspective reported by the EORTC QLQ-C30 and QLQ-CR29 will greatly enhance our understanding of quality of life of colorectal cancer survivors, for whom improved survival has been observed but subjective well-being has rarely been addressed. The combination of cancer core questionnaire and site specific module provides an effective way to measure quality-of-life status with excellent sensitivity and specificity, which in turn will facilitate colorectal cancer therapy and enhance comprehensive outcomes research.

## Abbreviations

ECOG: Eastern Cooperative Oncology Group
EORTC: European Organisation for Research and Treatment of Cancer
